# Predictive value of trauma scoring systems for mortality and intensive care outcomes among trauma patients: a study from central Iran

**DOI:** 10.1186/s12245-026-01191-4

**Published:** 2026-03-27

**Authors:** Naeimeh Heiranizadeh, Marzieh Azimizadeh, Seyedeh Mehrnoosh Mirvakili, Abdolhamid Amooee, Parisa Rahimian, Hamidreza Mohammadi

**Affiliations:** 1 Department of Surgery, Shahid Sadoughi University of Medical Sciences, Yazd, Iran; 2 Yazd Cardiovascular Research Center, Non-Communicable Diseases Research Institute, Shahid Sadoughi University of Medical Sciences, Afshar Hospital, Jomhouri Blvd, Yazd, Iran; 3 School of Medicine, Islamic Azad University of Medical Sciences, Yazd, Iran

**Keywords:** Glasgow coma scale, Injury severity score, Revised trauma score, Road traffic injury, Trauma

## Abstract

**Background:**

Rapid and accurate assessment of trauma severity is essential for guiding clinical decisions and improving patient outcomes. Although several trauma scoring systems exist, their practicality and predictive performance in real-world emergency settings remain under investigation. This study aimed to evaluate and compare the predictive value of three rapidly assessable trauma scoring systems, the Glasgow Coma Scale (GCS), Revised Trauma Score (RTS), and Injury Severity Score (ISS), for in-hospital mortality, intensive care unit (ICU) admission, and prolonged ICU stay among trauma patients in central Iran.

**Methods:**

This retrospective study analyzed 1,812 trauma patients admitted to Shahid Rahnemoun Hospital, Yazd, Iran, from April 2018 to May 2019. Demographic, clinical, and outcome data were collected from medical records. Logistic regression and receiver operating characteristic (ROC) curve analyses were used to assess the predictive performance of GCS, RTS, and ISS for trauma outcomes.

**Results:**

Road traffic injuries (RTIs) were the leading cause of trauma (60.6%), followed by falls (23.2%). The overall mortality rate was 1.6%, and 15.9% required ICU admission. ISS showed the strongest association with mortality (OR = 1.15; 95% CI: 1.10–1.20; *p* < 0.01), ICU admission (OR = 1.26; 95% CI: 1.23–1.30), and prolonged ICU stay (OR = 1.16; 95% CI: 1.13–1.20). ROC analysis confirmed ISS as the most accurate predictor across all outcomes: AUC = 0.948 (95% CI: 0.92–0.97) for mortality, 0.877 (95% CI: 0.85–0.90) for ICU admission, and 0.933 (95% CI: 0.91–0.95) for prolonged ICU stay. GCS and RTS also demonstrated acceptable predictive ability, particularly for prolonged ICU stay (AUC = 0.906 and 0.882, respectively).

**Conclusion:**

ISS exhibited superior predictive accuracy for mortality, ICU admission, and prolonged ICU stay compared with GCS and RTS. All three systems proved clinically useful, with ISS recommended for well-equipped trauma centers and GCS/RTS for rapid triage in resource-limited settings.

## Introduction

Trauma is a major global public health problem. There is increasing concern about the contribution of trauma to the burden of disease, as it is a leading cause of disability and death [1]. Trauma is the most common cause of death during the first four decades of life [2]. Traumatic events account for approximately 10% of global mortality and have a substantial impact on disability-adjusted life-years (DALYs) [3].

In Iran, road traffic injuries (RTIs) are the second leading cause of death. By 2030, RTIs may rank fifth among causes of mortality in developing countries [4]. Mortality from RTIs, especially among adolescents, requires increased attention [5]. One of the primary goals of emergency care is to reduce this burden and support timely decision-making in acute conditions [6].

In recent years, governmental policies have increasingly focused on preventive strategies to reduce mortality and severe complications. Given that multiple factors influence the mechanism of trauma and severity of trauma, developing treatment protocols based on local data is essential [7]. Evaluating epidemiological patterns and identifying predictors of mortality and critical care outcomes are crucial for optimizing trauma management and resource allocation. Trauma scoring systems are utilized to support efficient and accurate clinical decision-making. These systems are generally categorized as anatomical, physiological, or combined, each providing a different perspective on injury severity and patient prognosis [8, 9]. Despite ongoing advancements in developing new trauma scoring systems, a persistent challenge remains: their practical applicability for physicians in real-world emergency settings. In the fast-paced and resource-limited environment of emergency departments (EDs), the usefulness of a scoring system depends on how quickly and easily it can be calculated and interpreted at the bedside. Therefore, this study aimed to evaluate the predictive performance of three rapidly assessable trauma scoring systems included, Glasgow Coma Scale (GCS), Revised Trauma Score (RTS), and Injury Severity Score (ISS) for predicting in-hospital mortality and intensive care outcomes among trauma patients.

## Methods

### Study design and patient population

The retrospective observational study was conducted on all patients admitted following injury to the ED of Shahid Rahnemoun Hospital, affiliated with Shahid Sadoughi University of Medical Sciences (Yazd, Iran), a second-level trauma center in central Iran, between April 2018 to May 2019.

The study population included all trauma patients who presented to the ED with at least one traumatic injury, with diagnoses coded according to the International Classification of Diseases, 10th Revision (ICD-10) [10]. Patients who were admitted to relevant inpatient departments after initial management were included. Patients who left before receiving treatment or had incomplete medical records were excluded. The primary outcomes were in-hospital trauma-related mortality, intensive care unit (ICU) admission, and prolonged ICU stay (≥ 14 days).

### Data collection

Data were collected from patients’ medical files using a standardized checklist. The Collected data included patient age, sex, Body Mass Index (BMI), systolic blood pressure (SBP), respiratory rate (RR), injury location, area of injury, mechanisms of trauma, and trauma scores. Mechanisms of trauma were categorized into four groups: RTIs, falls, cut and/or stab injuries, and blunt trauma.

Trauma scores, including GCS, ISS, and RTS, were determined by physicians at the earliest possible time after patient arrival. The GCS was used to evaluate the level of consciousness based on three parameters: eye opening, verbal response, and motor response. The total score ranges from 3 to 15, with higher scores indicating a better neurological status. The ISS is a numerical scoring system used to assess the overall trauma severity. The ISS is calculated by assessing the Abbreviated Injury Scale (AIS) severity code to each of the six body regions and selecting the highest AIS score in the three most severely injured body regions. AIS scores range from 1 (minor injury) to 6 (maximum severity). The highest AIS scores for the three most severely injured regions are squared and summed to calculate the ISS. The ISS is calculated as follows: ISS= ${\mathrm{A}\mathrm{I}\mathrm{S}}_{1}^{2}$+${\mathrm{A}\mathrm{I}\mathrm{S}}_{2}^{2}$+${\mathrm{A}\mathrm{I}\mathrm{S}}_{3}^{2}$.

The total ISS ranges from 1 to 75. If any of the three highest scores is a 6 (the most severe injury), the ISS is set to 75 to indicate a critical injury [11]. The RTS is a physiological scoring system that includes the GCS, SBP, and RR. It is calculated as follows: RTS = (GCS code × 0.9368) + (SBP code × 0.7326) + (RR code × 0.2908) [12, 13].

### Ethics approval and consent to participate

This study was approved by the local ethics committee of Shahid Sadoughi University of Medical Sciences (IR.SSU.MEDICINE.REC.1399.012) and conducted based on the Declaration of Helsinki on medical research. Informed consent was obtained from all eligible individuals.

### Statistical analysis

Data were analyzed using SPSS version 26 (IBM SPSS Statistics, Chicago, IL, USA). Quantitative variables were expressed as mean ± standard deviation, while qualitative variables were expressed as frequency and percentage. Independent t-tests were applied to compare quantitative variables between two groups, while one-way analysis of variance (ANOVA) was used for comparisons among more than two groups. Associations between qualitative variables were assessed using the chi-square test. Univariate and multivariate binary logistic regression analyses were conducted to explore the relationships between trauma scores (ISS, GCS, and RTS) and patient outcomes, including mortality, ICU admission, and prolonged ICU stay. The results are presented as odds ratios (OR) with 95% confidence intervals (CI). Receiver operating characteristic (ROC) curve analysis was performed to assess the diagnostic performance of trauma scores, with optimal cut-off thresholds determined for predicting mortality, ICU admission, and prolonged ICU stay. A two-tailed p-value ≤ 0.05 was considered statistically significant.

## Results

A total of 1812 trauma patients, with a mean age of 33.53 ± 21.46 years, were included in the study. The majority of patients (73.1%) were older than 18 years. Most of participants were male (79.6%). The mean GCS, ISS, and RTS scores were 14.16 ± 2.38, 6.26 ± 6.56, and 7.68 ± 2.50, respectively. The most common mechanism of trauma was RTIs, accounting for 60.6% of cases, followed by falls (23.2%), cut and/or stab injuries (8.5%), and blunt trauma (7.7%). Overall, 15.9% of patients required ICU admission, with a mean ICU stay of 9.27 ± 9.19 days. A prolonged ICU stay was observed in 3.6% of patients, while the in-hospital mortality rate was 1.6%. Most injuries occurred on roads and highways (62.8%). The head (30.6%), lower limbs (23.0%), and upper limbs (19.9%) were the most frequently affected anatomical regions. Multiple traumas were observed in 9.8% of cases Regarding the location of trauma, most injuries occurred on roads and highways (62.8%), followed by at home (23.4%), public places (7.7%), and workplaces (6.1%).

As shown in Table 1, trauma mechanisms were associated with significant differences in demographic and clinical characteristics. RTIs were the most common mechanism and primarily affected younger patients (mean age: 32.1 ± 19.2 years) and males (81.1%). In contrast, falls were more common among older patients (mean age: 38.3 ± 27.9 years) and were associated with a lower proportion of males (69.8%, *p* < 0.001). The distribution of injured body regions differed significantly according to the mechanism of trauma (*p* < 0.001). Regarding patient outcomes, ICU admission was significantly higher among RTIs patients (20.1%) compared to other trauma groups (*p* < 0.001). The mean ISS was highest among RTI cases (7.81 ± 7.43), while cut and/or stab injuries had the lowest scores (2.84 ± 3.22, *p* < 0.001). Similarly, GCS and RTS values indicated greater injury severity among RTI patients (*p* < 0.001). (Table 1)

**Table 1:** Comparison of demographic, clinical, and outcome characteristics among patients with different mechanisms of trauma

	RTIs (*N* = 1098)	Falls (*N* = 421)	Cut/stab (*N* = 154)	Blunt trauma (*N* = 139)	Total (*N* = 1812)	*p*-value
**General conditions**						
Age	32.12 ± 19.24	38.34 ± 27.85	31.16 ± 15.15	32.74 ± 19.74	33.53 ± 21.46	**< 0.001**
Sex, Male	891 (81.1)	294 (69.8)	141 (91.6)	116 (83.5)	1442 (79.6)	**< 0.001**
BMI (Kg/m^2^)	23.8 ± 3.3	23.77 ± 5.47	23.92 ± 3.55	23.73 ± 3.6	23.8 ± 3.95	0.97
**SBP (mmHg)**	124.39 ± 13.69	123.99 ± 15.03	123.81 ± 9.38	122.8 ± 13.83	124.13 ± 13.71	0.61
**RR (beats/min)**	13.86 ± 4.04	13.83 ± 2.22	13.71 ± 2.22	13.58 ± 2.18	13.82 ± 3.44	0.81
**Injury location**						
Road and highway	1083 (98.6)	18 (4.3)	9 (5.8)	28 (20.1)	1138 (62.8)	**< 0.001**
At home	3 (0.3)	307 (72.9)	69 (44.8)	45 (32.4)	424 (23.4)	
Workplace	3 (0.3)	35 (8.3)	50 (32.5)	22 (15.8)	110 (6.1)	
Public places	9 (0.8)	61 (14.5)	26 (16.9)	44 (31.7)	140 (7.7)	
**Area of injury**						
Head	345 (31.4)	151 (35.9)	10 (6.5)	48 (34.5)	554 (30.6)	**< 0.001**
Thoracic	24 (2.2)	9 (2.1)	3 (1.9)	4 (2.9)	40 (2.2)	
Abdominal	20 (1.8)	5 (1.2)	0 (0.0)	5 (3.6)	30 (1.7)	
Spine and pelvis	102 (9.3)	53 (12.6)	0 (0.0)	8 (5.8)	163 (9.0)	
Upper limb	134 (12.2)	79 (18.8)	109 (70.8)	38 (27.3)	360 (19.9)	
Lower limb	287 (26.1)	99 (23.5)	15 (9.7)	16 (11.5)	417 (23.0)	
External	37 (3.4)	6 (1.4)	17 (11.0)	11 (7.9)	71 (3.9)	
Multiple trauma	149 (13.6)	19 (4.5)	0 (0.0)	9 (6.5)	177 (9.8)	
**Outcomes**						
ICU admission	221 (20.1)	51 (12.1)	1 (0.6)	16 (11.5)	289 (15.9)	**< 0.001**
Length of ICU stay (days)	9.78 ± 9.75	7.55 ± 6.87	8 ± 0	7.81 ± 7.38	9.27 ± 9.19	0.41
Prolonged ICU stay	54 (24.4)	9 (17.6)	0 (0.0)	3 (18.8)	66 (3.6)	0.67
Mortality	20 (1.8)	8 (1.9)	0 (0.0)	1 (0.7)	29 (1.6)	0.3
**Trauma scores**						
ISS	7.81 ± 7.43	7.08 ± 7.5	2.84 ± 3.22	4.83 ± 4.98	6.99 ± 7.17	**< 0.001**
GCS	13.84 ± 2.79	14.57 ± 1.55	15 ± 0	14.45 ± 1.83	14.15 ± 2.38	**< 0.001**
RTS	7.28 ± 0.74	7.46 ± 0.39	7.55 ± 0	7.43 ± 0.43	7.36 ± 0.62	**< 0.001**

Data presented as mean ± standard deviation or number (%). Abbreviations: RTIs, Road traffic injuries; BMI, Body Mass Index; SBP, systolic blood pressure; RR, Respiratory Rate; GCS, Glasgow Coma Scale; ISS, Injury Severity Score; RTS, Revised Trauma Score

Table 2 presents a comparison of trauma scores across different patient outcomes. The mean ISS score was significantly higher in patients who died compared with survivors (18.72 ± 9.58 vs. 6.06 ± 6.31, *p* < 0.001), whereas the mean GCS (7.93 ± 4.64 vs. 14.26 ± 2.20, *p* < 0.001) and RTS (5.81 ± 1.41 vs. 7.66 ± 0.58, *p* < 0.001) were significantly lower. Similarly, patients who required ICU admission or experienced prolonged ICU stay had higher ISS values and lower GCS and RTS scores. (Table 2)

**Table 2:** Comparison of trauma scores according to patient outcomes

	Mortality		*P*-value	ICU admission		*P*-value	Prolonged ICU stay		*P*-value
	Yes	No		Yes	No		Yes	No	
ISS	27.76 ± 17.45	6.65 ± 6.36	< 0.001	16.4 ± 10.63	5.2 ± 4.46	< 0.001	21.83 ± 11.7	14.8 ± 9.76	< 0.001
GCS	7.83 ± 4.5	14.26 ± 2.2	< 0.001	10.43 ± 4	14.9 ± 0.7	< 0.001	7.67 ± 3.85	11.25 ± 3.8	< 0.001
RTS	5.68 ± 1.47	7.38 ± 0.56	< 0.001	6.46 ± 1.16	7.53 ± 0.17	< 0.001	5.7 ± 1.19	6.68 ± 1	< 0.001

Table 3 presents the results of crude and fully adjusted logistic regression models. Higher ISS was significantly associated with increased odds of mortality (OR = 1.12; 95% CI: 1.07–1.16; *p* < 0.05) and ICU admission (OR = 1.25; 95% CI: 1.22–1.28; *p* < 0.05). In contrast, higher GCS (OR = 0.65; 95% CI: 0.58–0.72; *p* = 0.01) and RTS (OR = 0.21; 95% CI: 0.14–0.31; *p* = 0.01) were associated with lower odds of mortality. Similarly, prolonged ICU stay was associated with higher ISS (OR = 1.08; 95% CI: 1.05–1.12; *p* < 0.05) and lower GCS (OR = 0.79; 95% CI: 0.72–0.85; *p* < 0.05) and RTS (OR = 0.48; 95% CI: 0.36–0.63; *p* < 0.05). (Table 3)

**Table 3:** Logistic regression analysis of trauma scores associated with patient outcomes

		ISS		GCS		RTS	
		OR (CI 95%)	p-value	OR (CI 95%)	p-value	OR (CI 95%)	p-value
Mortality	Crude	1.16 (1.15–1.20)	0.01	0.67 (0.61–0.73)	0.01	0.27 (0.21–0.36)	0.01
	Full adjust	1.15 (1.10–1.20)	0.01	0.63 (0.56–0.71)	0.01	0.23 (0.16–0.33)	0.01
ICU admission	Crude	1.27 (1.24–1.31)	0.01	0.41 (0.36–0.46)	0.01	0.037 (0.02–0.05)	0.01
	Full adjust	1.26 (1.23–1.30)	0.01	0.41 (0.36–0.46)	0.01	0.038 (0.02–0.06)	0.01
Prolonged ICU stay	Crude	1.17 (1.13–1.2)	0.01	0.62 (0.58–0.67)	0.01	0.21 (0.17–0.27)	0.01
	Full adjust	1.16 (1.13–1.2)	0.01	0.61 (0.57–0.66)	0.01	0.20 (0.15–0.26)	0.01

The results are expressed as Odds ratio (OR) and 95% confidence intervals (CI). “Crude” refers to unadjusted model. “Full adjust” refers to model adjusted for Age, Sex, BMI, Injury locale, Area of injury, and Mechanism of trauma. *p* < 0.05 was considered statistically significant

**Fig. 1:**
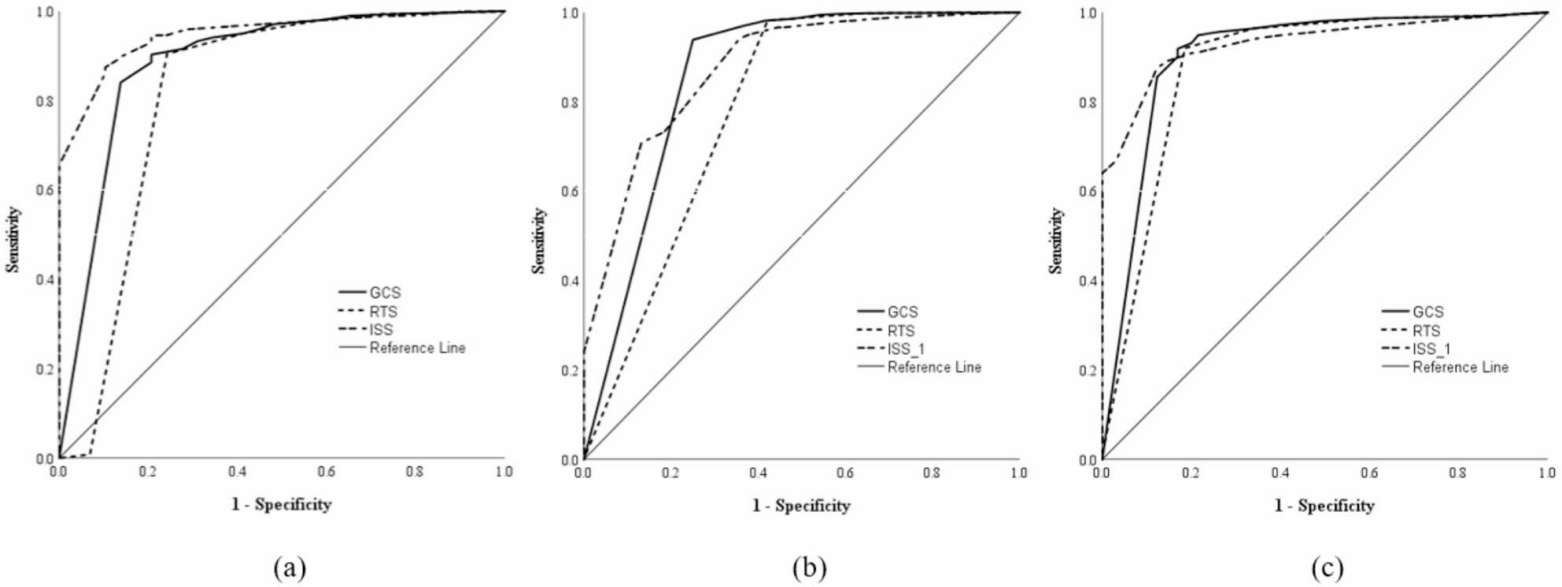
The receiver–operating characteristic (ROC) curve analysis of trauma scores for predicting mortality (**a**), ICU admission (**b**), and (**c**) prolonged ICU stay

The ROC curve analysis was performed to evaluate the predictive performance of trauma scores for trauma outcomes. For mortality prediction, the ISS area under the curve (AUC) was 0.94 (95% CI: 0.92–0.97) with a cut-off value of 12.5, sensitivity of 0.89, and specificity of 0.87. This was followed by GCS (AUC = 0.888, 95% CI: 0.81–0.96) and RTS (AUC = 0.816, 95% CI: 0.70–0.93). For ICU admission, ISS showed the highest predictive performance (AUC = 0.877, 95% CI: 0.85–0.90; cut-off = 9.5; sensitivity = 0.63; specificity = 0.94), followed by GCS (AUC = 0.858) and RTS (AUC = 0.783). For prolonged ICU stay, ISS had the highest AUC (0.933; 95% CI: 0.91–0.95; sensitivity = 0.86; specificity = 0.88), followed by GCS (AUC = 0.906) and RTS (AUC = 0.882). (Table 4; Fig. 1)

**Table 4:** Predictive performance of trauma scores based on ROC curve analysis for trauma outcomes

		AUC (95%CI)	Cut off	Sensitivity	Specificity
Mortality	ISS	0.948 (0.92–0.97)	12.5	0.89	0.87
	GCS	0.888 (0.81–0.96)	12.5	0.9	0.79
	RTS	0.816 (0.70–0.93)	6.71	0.9	0.76
ICU Admission	ISS	0.877 (0.85–0.90)	9.5	0.63	0.94
	GCS	0.858 (0.82–0.88)	14.5	0.93	0.75
	RTS	0.783 (0.74–0.82)	7.25	0.97	0.58
Prolonged ICU Stay	ISS	0.933 (0.91–0.95)	12.5	0.86	0.88
	GCS	0.906 (0.85–0.95)	10.5	0.94	0.78
	RTS	0.882 (0.82–0.93)	6.71	0.92	0.81

## Discussion

Trauma remains one of the leading causes of mortality and morbidity, especially in developing countries [14]. In the present study, RTIs were the most common mechanism of trauma, followed by falls. RTIs accounted for 60.6% of all cases and were associated with increased in-hospital mortality. The predominance of RTIs is consistent with previous studies indicating that Iran is among the countries where injuries, particularly those related to motor vehicle accidents, are major causes of death [15, 16].

In the present study, the mean age of patients was 33.5 years, and 79.6% were male. These findings are consistent with previous studies reporting that men and younger adults form are more frequently affected, likely due to higher mobility and occupational exposure [17–19]. The mean age was significantly higher in those who sustained falls. Mosenthal et al. [20] and Grossman et al. [21] reported that individuals who sustained fall-related injuries were, on average, older than those injured by other mechanisms. In another study, Báča et al. demonstrated that trauma injuries were more common among men, while falls were the leading cause of trauma in women [18]. Consistent with previous findings by Sterling et al. [22] and Yadollahi et al. [23], RTIs were associated with higher ISS values and lower GCS and RTS scores, suggesting greater injury severity and multisystem involvement. In contrast, cutting and stabbing injuries were associated with lower ISS values, suggesting more localized and less extensive anatomical damage.

It is well established that early and accurate assessment of trauma severity is associated with reduced mortality and disability [24]. In the present study, the prognostic performance of three widely used rapid trauma scoring systems for predicting mortality, ICU admission, and prolonged ICU stay was compared. The findings indicated that ISS had the highest predictive performance among the evaluated scoring systems. Despite advancements in trauma scoring systems, their ease of use and simplicity remain important but often underemphasized factors. Although newer scoring systems may offer greater accuracy, they are often more difficult for physicians to apply in critical situations. Therefore, simpler and more rapidly assessable scoring systems may be more practical in such settings.

Rapidly assessable trauma scoring systems facilitate timely and accurate clinical decision-making in trauma management. These systems can be applied both before patient transfer to a trauma center and during clinical decision-making upon arrival. They also assist in patient triage and management in the emergency department, including decisions regarding surgical intervention and communication with patients’ families about injury severity [25, 26].

The findings of the current study showed that all three scoring systems had acceptable performance in predicting trauma outcomes. However, the ISS demonstrated higher predictive performance for all evaluated outcomes compared with the physiological scoring systems. In contrast, physiological scoring systems showed relatively better performance in predicting prolonged ICU stay than other outcomes.

Several studies have compared the performance of trauma scoring systems in predicting clinical outcomes. Previous studies have reported that ISS is among the most effective scoring systems for predicting in-hospital mortality and injury severity. For instance, a study involving 200 trauma patients aged over 60 years reported an AUC of 0.963 for ISS, which was higher than those of RTS (0.947) and GCS [27]. Similarly, a retrospective study on 326 trauma patients admitted to the ICU demonstrated that ISS had the highest predictive performance for mortality (AUC = 0.82), compared with GCS (0.69) and RTS (0.74) [28]. A study based on the National Trauma Registry of Iran reported that ISS was the best predictor of in-hospital mortality and ICU admission compared with GCS and RTS [29]. Another study of 1,930 trauma cases in the Iranian population found that the ISS had the highest AUC among the three scoring systems [25]. Additionally, a study of 938 trauma patients under the age of 6 reported that ISS had the highest AUC for predicting mortality (0.975), followed by RTS (0.899) and GCS (0.864) [30].

In contrast, some previous studies demonstrated that GCS may provide better prediction trauma outcomes [31, 32]. For example, a study of 200 trauma patients admitted to the ICU reported that GCS had higher predictive performance than other scoring systems for adverse outcomes [33]. A prospective study also reported that the AUC of GCS for predicting in-hospital mortality was 0.91, which was higher than that of RTS, highlighting the importance of GCS in assessing neurological function and predicting mortality in trauma patients [34]. These findings suggests that GCS may be particularly useful in evaluating patients with suspected brain injuries. Additionally, a study of 1,410 trauma patients reported that RTS had the highest AUC (0.93) for predicting mortality, followed by TRISS (0.85), ISS (0.80), and GCS (0.75). These findings support the potential utility of RTS, particularly in situations where physiological stability is a primary concern [35].

Overall, despite variations in AUC values across different trauma scoring systems, each system appears to provide valuable information for predicting trauma outcomes. Therefore, the selection of an appropriate scoring system should be tailored to available resources and the clinical setting. In trauma centers with access to advanced diagnostic and imaging modalities, such as computed tomography (CT), ultrasonography, and radiography, ISS may be more useful for assessing injury severity. In contrast, in settings with limited diagnostic facilities or those located far from tertiary care centers, GCS and RTS may be more practical tools to support rapid clinical decision-making and patient triage, thereby facilitating timely identification and management of critically injured patients.

These findings suggest that integrating trauma scoring systems into routine triage protocols may improve early risk stratification and support timely clinical decision-making. Standardized training of emergency personnel in the application of these tools, along with their incorporation into clinical workflows, may reduce variability in patient assessment and enhance the efficiency of trauma care. Such approaches may ultimately contribute to improved patient outcomes through more appropriate resource allocation and timely intervention.

### Limitations

This study has several limitations that should be acknowledged. First, the retrospective and single-center design may limit the generalizability of the findings to other trauma populations or healthcare settings with different referral patterns, resources, and trauma care protocols. Second, the trauma scores and clinical criteria were evaluated by different physicians during various work shifts, which may have introduced interobserver variability. Moreover, inconsistencies or errors in data recording in the medical files may have affected the accuracy of the collected information. Third, although the study adjusted for key demographic and clinical variables, potential confounders such as pre-hospital care, time to hospital arrival, comorbidities, and injury mechanism details were not available and may have influenced outcomes. Fourth, long-term outcomes such as post-discharge mortality, disability, or quality of life were not evaluated; hence, the predictive validity of the trauma scores beyond hospitalization remains unknown. Finally, this study included only three rapidly assessable trauma scoring systems. Future multicenter, prospective studies with larger and more diverse populations are needed to validate and extend these findings.

## Conclusion

The findings highlight the clinical utility of ISS, GCS, and RTS as rapid and practical tools for triaging trauma patients and guiding early management in emergency settings. The present study demonstrated that among rapidly assessable trauma scoring systems, ISS had the higher predictive performance for mortality, ICU admission, and prolonged ICU stay, followed by GCS and RTS.

Future studies should focus on developing hybrid or AI-assisted models that integrate multiple trauma parameters, as well as simple and user-friendly clinical tools for rapid bedside use, to enhance real-time decision-making and outcome prediction in trauma care.

## Data Availability

The datasets used and/or analysed during the current study are available from the corresponding author on reasonable request.
